# Supplementary material for: Farrerol directly activates the deubiqutinase UCHL3 to promote DNA repair and reprogramming when mediated by somatic cell nuclear transfer
Source: Nat Commun. 2023 Apr 3;14:1838. doi: 10.1038/s41467-023-37576-9 (PMC10070447; doi:10.1038/s41467-023-37576-9)
Supplement: Supplementary file 1 — Supplementary information [file 41467_2023_37576_MOESM1_ESM.pdf]

Supplementary Figures

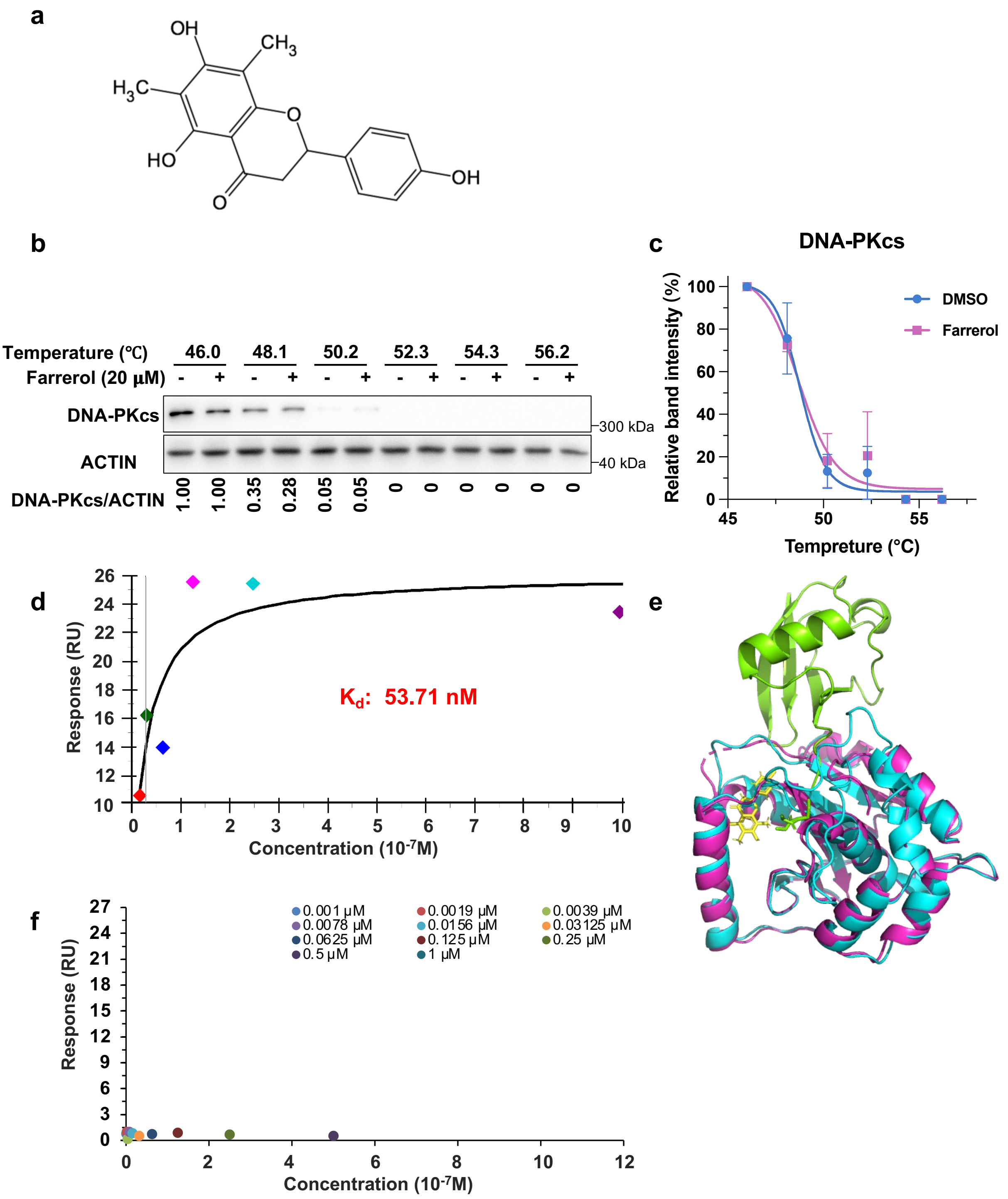

Supplementary Figure 1 Determination of the binding between farrerol and DNA-PKcs or UCHL3.

(a) The chemical structure of farrerol. **(b)** Representative immunoblots for the CETSA assay carried out in HEK293 cells treated with 20 µM farrerol (+) or DMSO (–). Western blots were developed using an anti-DNA-PKcs antibody. **(c)** CETSA curves of DNA-PKcs in HEK293 cells were determined in the absence and presence of farrerol. Each band intensity for UCHL3 was normalized with respect to that obtained at 46 °C. The graphs are representative of three independent experiments. **(d)** Another SPR analysis of farrerol binding to recombinant UCHL3. **(e)** Overlay of farrerol in complex with UCHL3 (UCHL3 in blue) and UCHL3-UbVME complex (UCHL3 in magenta with Ubiquitin in green), together with the 2D chemical structures of farrerol in yellow. **(f)** UCHL3-2A fails to bind farrerol. UCHL3-2A (R215A K187A) was immobilized on a BIAcore CM5 sensor chip, followed by the analysis of its interaction with farrerol by an SPR assay. Data are presented as mean values ± s.e.m. (**c**). Source data are provided as a Source Data file.

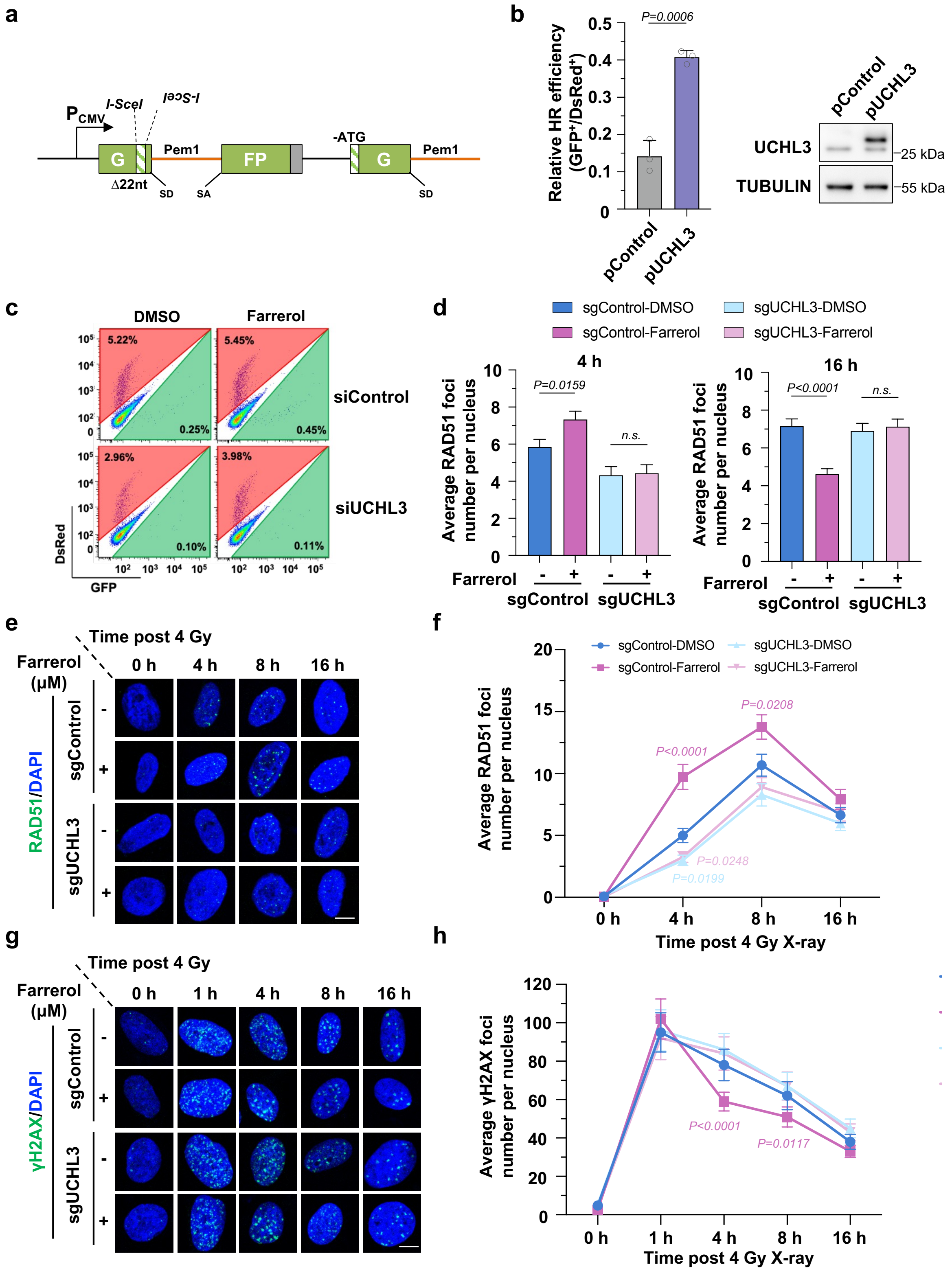

Supplementary Figure 2 Farrerol promotes HR repair dependent on UCHL3.

(a) Diagram of the HR reporter as previously described [36]. **(b)** Effect of UCHL3 overexpression on HR efficiency (n=3). Western blot analysis of UCHL3 overexpression in HR reporter cells is shown in the right panel. **(c)** Representative FACS traces are shown with siRNA against the control and UCHL3. **(d)** Quantification of RAD51 foci at 4 h and 16 h post IR at 2 Gy, and at least 40 cells were counted for each group. **(e)** Representative images of RAD51 foci at indicated times after irradiation (4 Gy) are presented. Scale bar: 5 μm. **(f)** Quantification of RAD51 foci number in the control and UCHL3-KO cells at indicated time points post 4 Gy IR, and at least 40 cells were counted for each group. **(g)** Representative images of γH2AX foci at indicated times after irradiation (4 Gy) are presented. Scale bar: 5 μm. **(h)** Quantification of γH2AX foci number in the control and UCHL3-KO cells at indicated time points post 4 Gy IR, and at least 40 cells were counted for each group. Data are presented as mean values ± s.d. (**b**). Data are presented as mean values ± s.e.m. (**d**, **f**, **h**). Statistical significance was calculated with a two-tailed unpaired t-test (**b**, **d**, **f**, **h**). n.s. not significant. Source data are provided as a Source Data file.

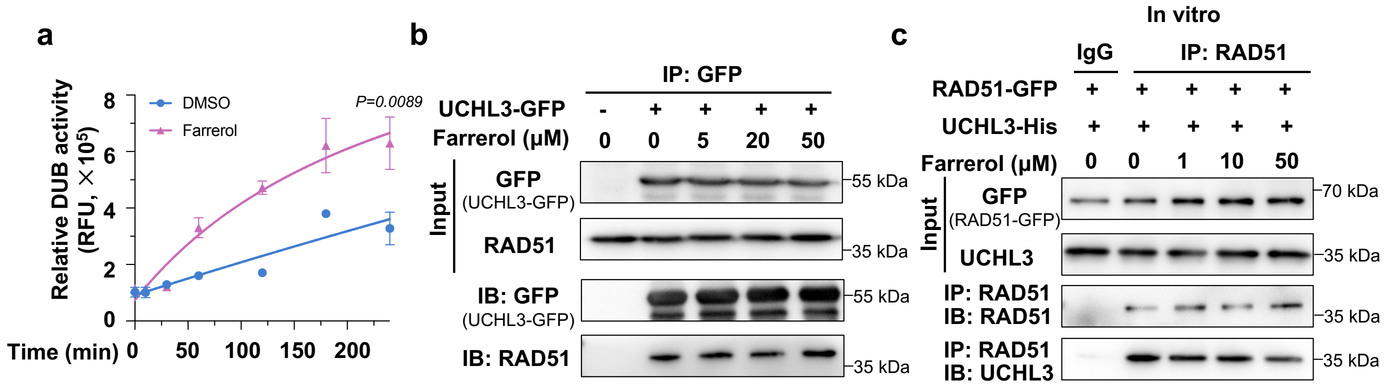

Supplementary Figure 3 Interactions between UCHL3 and RAD51 after farrerol treatment.

(a) Analysis of UCHL3 enzymatic activity in the presence or absence of 0.1 μM farrerol using a Ub-CHOP2-reporter deubiquitylation assay (n=3). **(b)** Co-IP in HEK293 cells transfected with UCHL3-GFP and treated with DMSO or farrerol for 24 h. **(c)** In vitro co-IP assay of the interaction between UCHL3 and RAD51 treated with farrerol. Purified UCHL3-His and RAD51-GFP were incubated in a cell-free system, incubated with diluted concentrations of farrerol, and then subjected to IP with antibodies that recognized RAD51, followed by Western blot analysis. Data are presented as mean values ± s.e.m. (**a**). Statistical significance was calculated with a two-tailed unpaired t-test (**a**). Experiments were repeated three times independently with similar results; data of one representative experiment are shown (**b**,**c**). Source data are provided as a Source Data file.

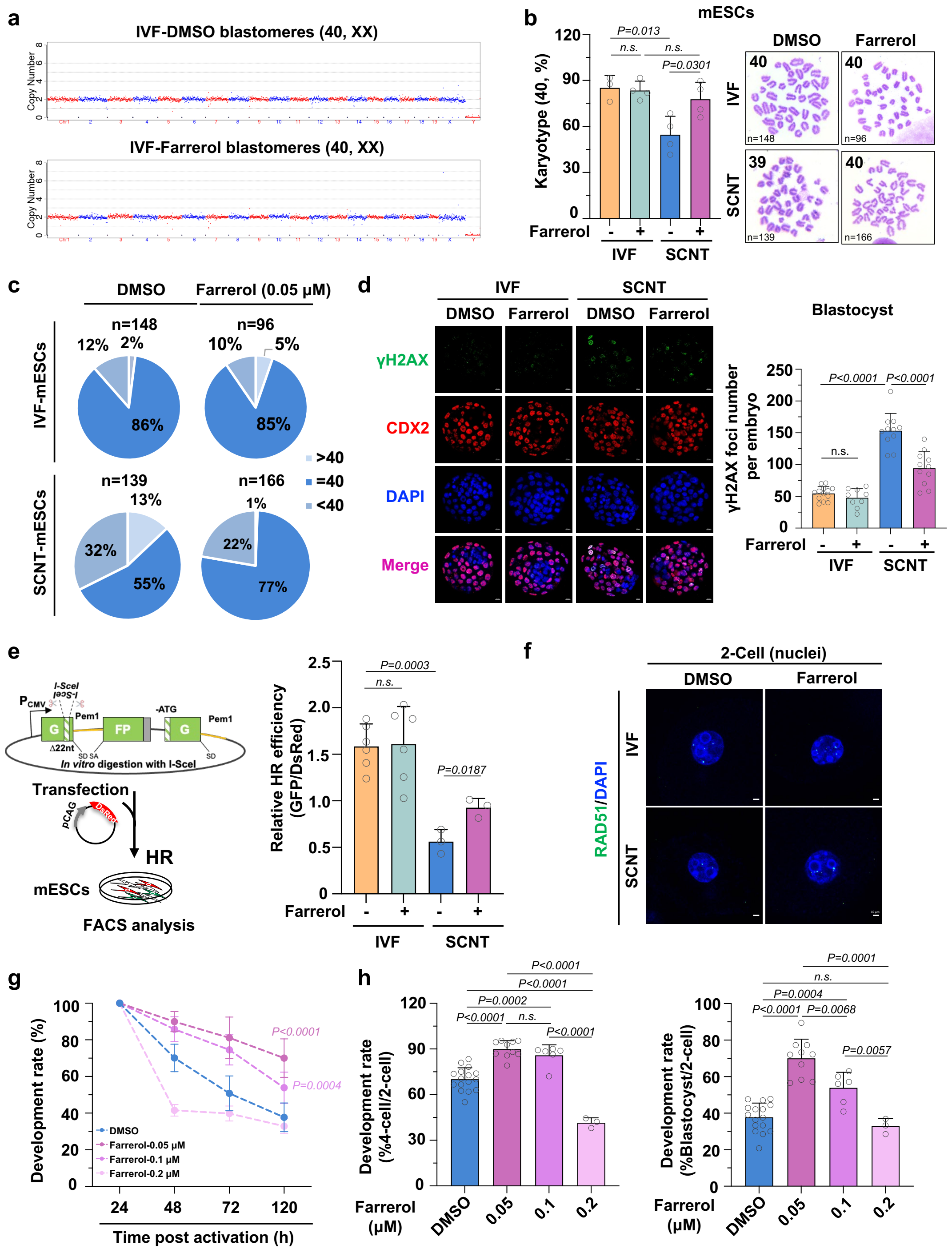

Supplementary Figure 4 Farrerol promotes the developmental progression of SCNT embryos.

(a) Representative images of copy number variation (CNV) analysis in IVF embryos treated with or without farrerol. 1-2 blastomeres from embryos at the 4-cell stage were randomly picked for the examination. **(b)** The karyotype statistic (left) and representative images (right) of mESCs derived from IVF or SCNT treated with DMSO or 0.05 µM farrerol. Each dot indicates a cell line (n=3-4). **(c)** Pie charts showing the percentage of fewer (<40), more (>40), or normal (=40) chromosome counts in indicated groups. **(d)** Immunofluorescence of blastocysts stained with anti-γH2AX (green) and anti-CDX2 (red) antibodies and DAPI (blue). The number of γH2AX foci is counted in the right (n=9-13 blastocysts). Scale bar: 10 µm. **(e)** Schematic depiction of the HR repair assay. The HR reporter was linearized by I-SceI endonuclease in vitro to mimic DSBs. The purified linearized HR reporters along with the pDsRed-N1 vector for normalizing transfection efficiency were transfected into mESCs (n=3-6). FACS analysis was performed at 48 h post transfection. **(f)** Representative images of IVF and SCNT 2-cell embryos stained with anti-RAD51 (green) antibodies and DAPI (blue) related to Fig. 4f. Scale bar: 10 µm. **(g)** Development rate of SCNT embryos treated with different concentrations of farrerol (n=3-16 embryos). **(h)** Development rate of 4-cell embryos (left) and hatching blastocysts at E4.5 (right) treated with the indicated concentration of farrerol (n=3-16 embryos). Data are presented as mean values ± s.d. (**b**, **d**, **e**, **g**, **h**). Statistical significance was calculated with a two-tailed unpaired t-test (**b**, **d**, **e**, **g**, **h**). n.s. not significant. Source data are provided as a Source Data file.

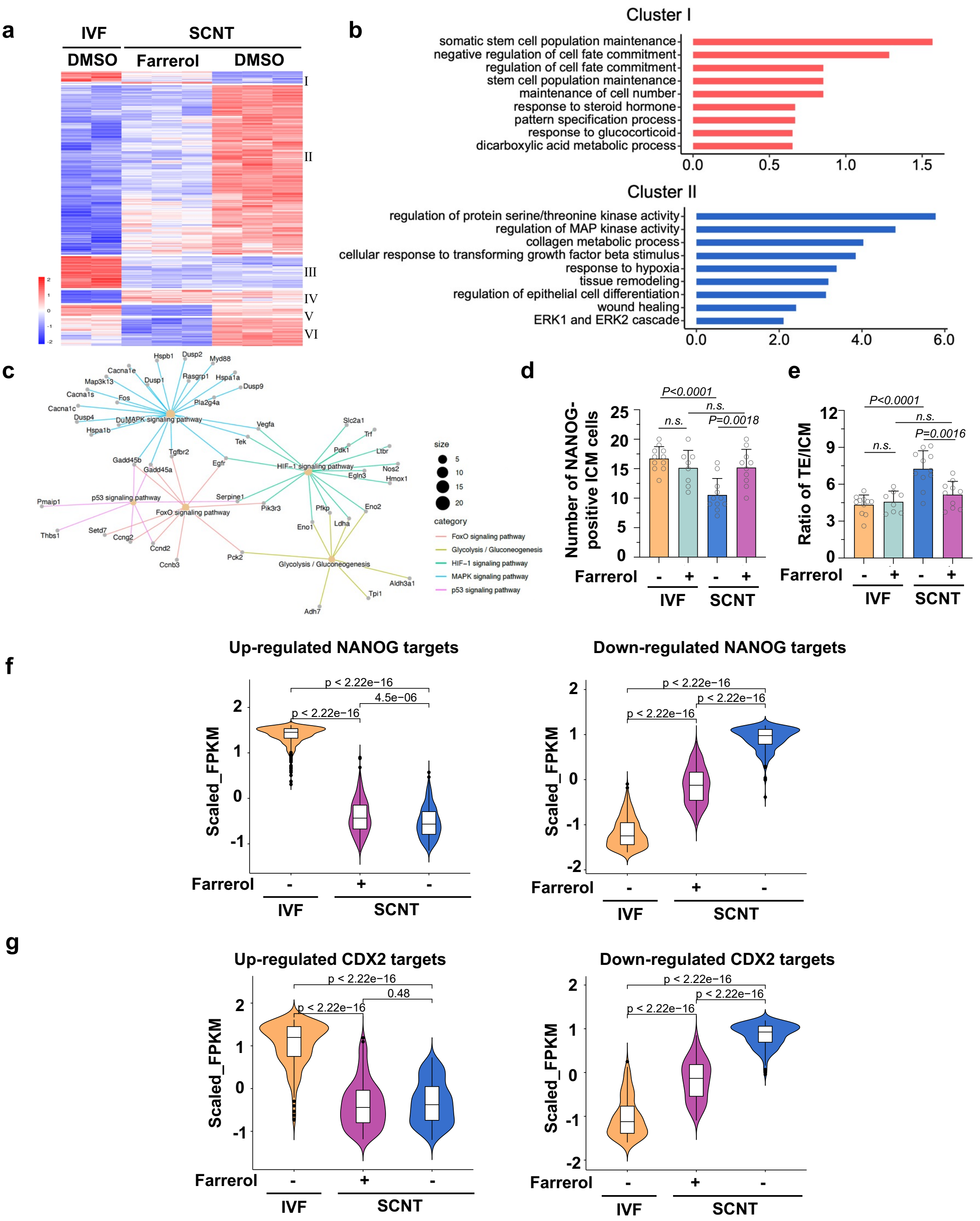

Supplementary Figure 5 The farrerol-treated group of SCNT embryos exhibits a similar gene transcription pattern to the IVF group. **(a)** Heatmap showing the hierarchical clustering of differentially expressed genes in morula stage embryos from indicated groups. **(b)** Gene Ontology analysis of the gene Cluster I and Cluster II in the heatmap related to Supplementary Figure 5a. **(c)** KEGG pathway analysis of the genes in Cluster I and Cluster II. The 5 pathways with the lowest adjusted p value and related genes are shown. **(d)** Plot showing the number of NANOG-positive ICM cells in blastocysts in indicated groups related to Fig. 5e (n=8-11 blastocysts). **(e)** The ratio of TE/ICM in indicated embryos related to Fig. 5e and Supplementary Figure 5d (n=8-11 embryos). **(f)** Violin plot showing the expression of NANOG targets. The target gene list is from CHEA Transcription Factor Targets, the 1000 NANOG targets with the greatest foldchange between IVF and SCNT were selected and divided into two groups: up-regulated in IVF (left) (n=660) and down-regulated in IVF (right) (n=340). **(g)** Violin plot showing the expression of CDX2 targets. The target gene list is from CHEA Transcription Factor Targets, the 400 CDX2 targets with the greatest foldchange between IVF and SCNT were selected and divided into two groups: up-regulated in IVF (left) (n=217) and down-regulated in IVF (right) (n=183). The central band indicated the median and the box bound indicated the first and third quartiles, and the whiskers indicated ±1.5× interquartile range (**f, g**). Data are presented as mean values ± s.d. (**d, e**). Statistical significance was calculated with a two-tailed unpaired t-test (**c-g**). n.s. not significant. Source data are provided as a Source Data file.

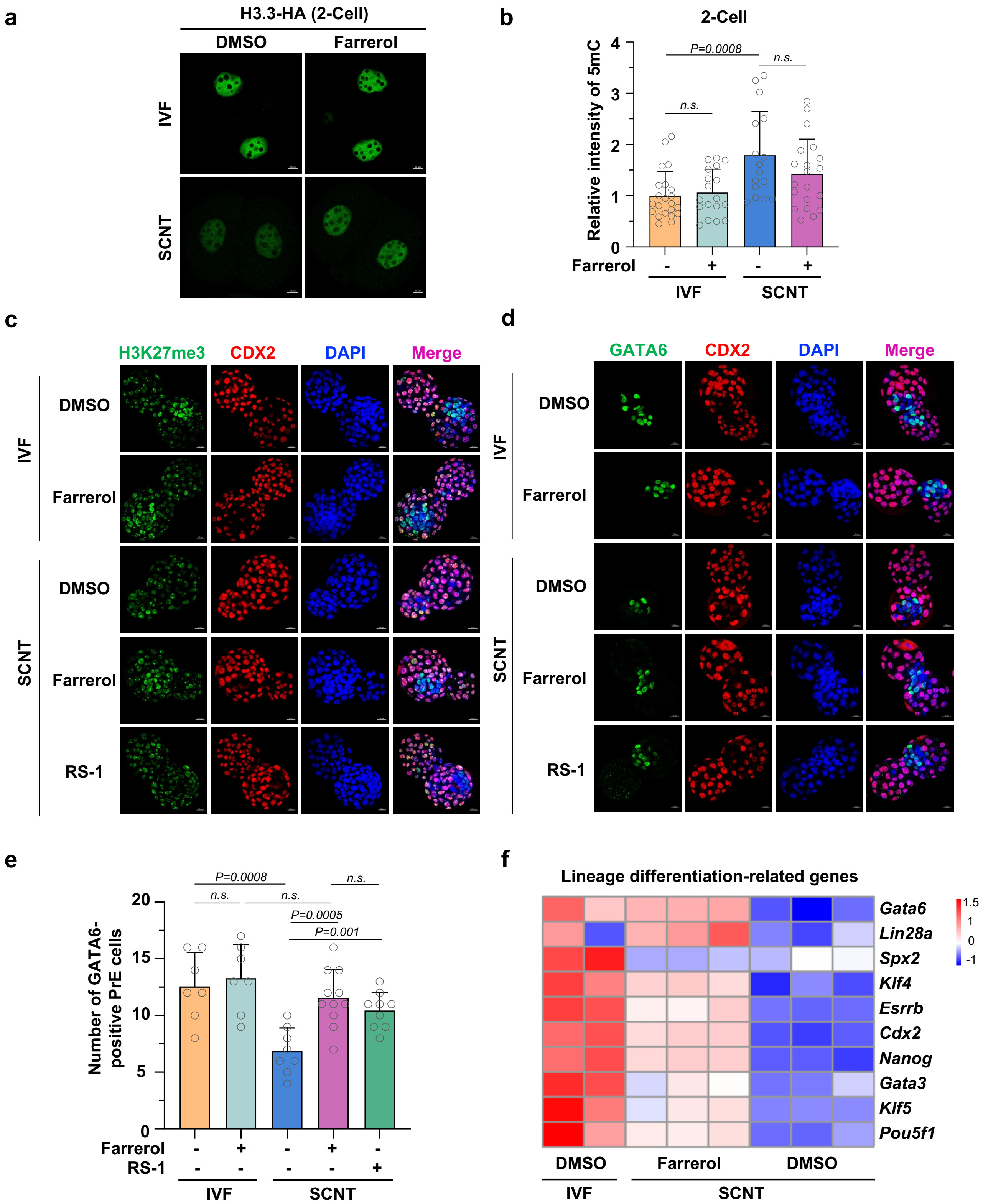

Supplementary Figure 6 Farrerol improves the differentiation potential of SCNT embryos.

(a) Representative images of 2-cell embryos in indicated groups stained with an anti-HA antibody related to Fig. 5g. The HA antibody binds to the HA tag carried by the exogenously injected histone variant H3.3 mRNA (100 ng/μL). Scale bar: 10 μm. **(b)** The relative 5mC intensity per nucleus of 2-cell embryos in indicated groups with or without farrerol treatment (n=16-22 embryos). **(c)** Representative images of immunofluorescence of blastocysts stained with anti-H3K27me3 (green) and anti-CDX2 (red) antibodies and DAPI (blue) related to Fig. 5h. Scale bar: 20 μm. **(d)** Immunofluorescence of blastocysts stained with anti-GATA6 (green) and anti-CDX2 (red) antibodies and DAPI (blue). Scale bar: 20 μm. **(e)** Plot showing the number of GATA6-positive primitive endoderm (PrE) cells with 0.05 μM farrerol or 10 μM RS-1 treatment (n=7-11 cells). **(f)** Heatmap showing the relative expression of genes related to ICM and TE differentiation. Data are presented as mean values ± s.d. (**b, e**). Statistical significance was calculated with a two-tailed unpaired t-test (**b, e**). n.s. not significant. Experiments were repeated three times independently with similar results; data of one representative experiment are shown (**c, d**). Source data are provided as a Source Data file.

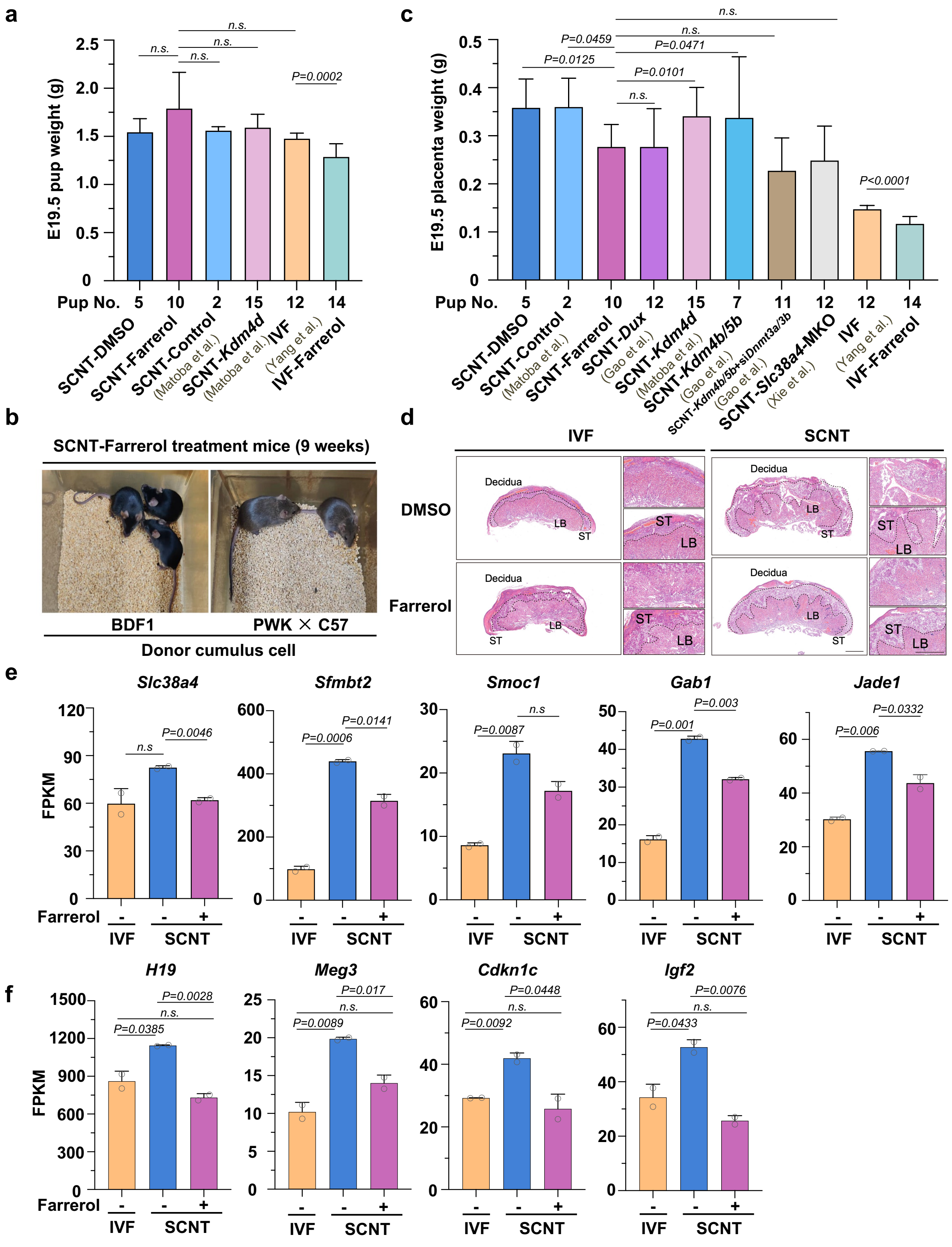

Supplementary Figure 7 Effect of farrerol on SCNT offspring at birth.

(a) Weight comparison of the full-term E19.5 pups generated by indicated groups (n=5-14 pups). Several data are cited from published studies as indicated [16, 17]. **(b)** Representative images of the farrerol-treated SCNT mice using BDF1 (C57×DBA2) and BPF1(C57×PWK) as donors. **(c)** Weight comparison of the placentae from the E19.5 pups generated by farrerol-treated SCNT embryos and control SCNT embryos (n-5-14 pups). Several data are cited from published studies as indicated [15-17, 50]. **(d)** Paraffin section of E19.5 placenta stained with hematoxylin and eosin from the IVF or SCNT embryos treated with DMSO or farrerol. Scale bar: 1 mm. ST: spongiotrophoblast layer; LB: labyrinthine layer. **(e)** Expression of representative non-canonical imprinted genes in extraembryonic ectoderm cells of E7.5 IVF, and E7.5 SCNT embryos with or without farrerol treatment (n=2). **(f)** Expression of indicated canonical imprinted genes in extraembryonic ectoderm cells of E7.5 IVF, and E7.5 SCNT embryos with or without farrerol treatment (n=2). Data are presented as mean values ± s.d. (**a**, **c**, **e**, **f**). Statistical significance was calculated with a two-tailed unpaired t-test (**a**, **c**, **e-f**). n.s. not significant. Experiments were repeated three times independently with similar results; data of one representative experiment are shown (**d**). Source data are provided as a Source Data file.

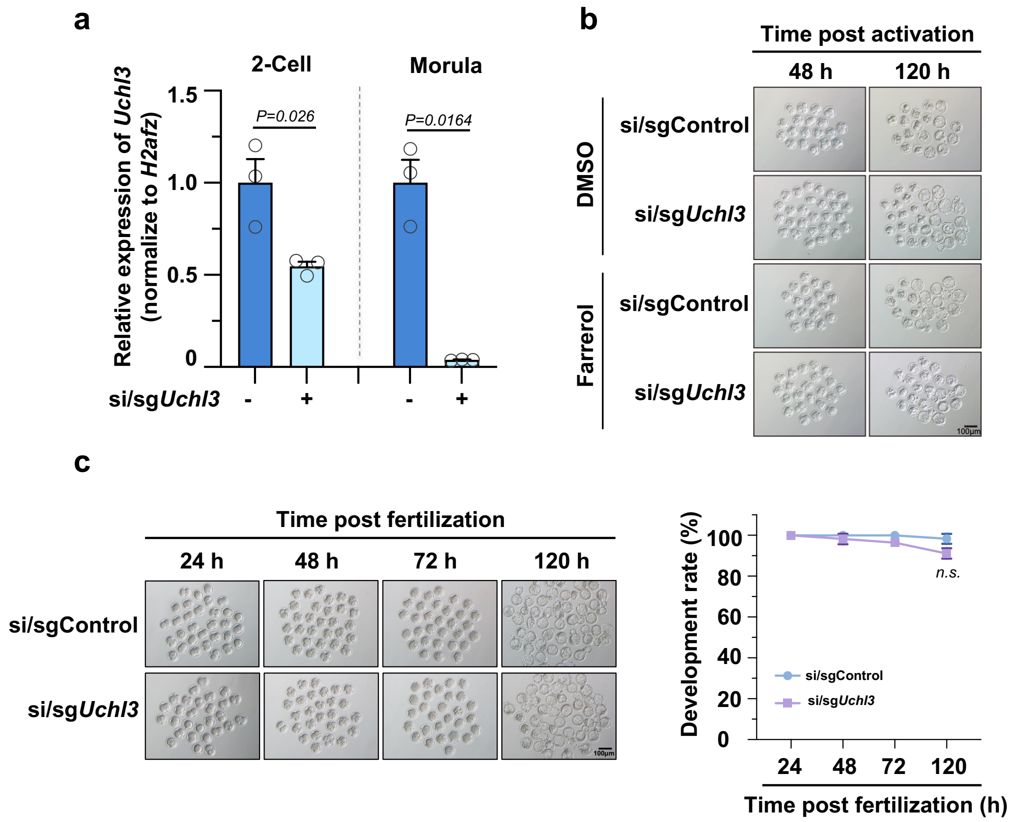

Supplementary Figure 8 Loss of *Uchl3* impairs the farrerol-mediated improvement in SCNT embryo development.

(a) RT–qPCR analysis showing the relative expression of *Uchl3* in 2-cell and morula embryos in indicated groups. *H2afz* (H2A family member Z) was used as an endogenous control. Biological replicates (n = 3) were performed. **(b)** Representative images of the SCNT embryos at the 4-cell stage and blastocyst stage by indicated manipulations, as related to Fig. 6b. **(c)** Representative images (left) and the development rate (right) of IVF embryos after *Uchl3* depletion. Depletion of *Uchl3* by targeting siRNAs and sgRNA did not greatly influence the preimplantation development of IVF embryos (n=2 independent experiments, at least 28 embryos were analyzed for each experiment). Scale bar: 100 μm, Data are presented as mean values ± s.d. (**a, c**). Statistical significance was calculated with a two-tailed unpaired t-test (**a**, **c**). n.s. not significant. Experiments were repeated two times independently with similar results; data of one representative experiment are shown (**b**, **c**). Source data are provided as a Source Data file.

Supplementary Table 1 Preimplantation Development of IVF and SCNT embryos

Embryo type	mRNA injection	Treatment	No. of replicates	No. of reconstructed 2-cell embryos	%4-cell per 2-cell ± SD	%morula per 2-cell ±SD	%blastocyst per 2-cell ±SD
IVF	/	Control-DMSO (1000×, maintain)	3	60	100±0.00	96.11±2.83	93.44±1.23
	/	Mirin (50 μM, maintain)	4	58	0	0	0
	/	Mirin (50 μM, 16 h)	2	45	96.15±3.85	92.31±7.69	81.78±2.83*
	/	Farrerol (0.05 μM, maintain)	3	65	100±0.00	98.25±2.48	92.45±1.62
	/	Farrerol (0.05 μM, 16 h)	2	40	100±0.00	100±0.00	100±0.00**
SCNT	/	Control-DMSO (1000×, maintain or 16 h)	16	346	70.16±7.28	50.80±9.19	37.69±7.62
	/	Mirin (50 μM, maintain)	4	0	0	0	0
	/	Mirin (50 μM, 16 h)	3	53	23.21±11.04***	13.84±9.00***	7.14±6.73***
	/	Farrerol (0.05 μM, maintain)	2	38	63.06±1.94	0	0
	/	Farrerol (0.05 μM, 16 h)	10	217	89.95±5.20***	81.09±10.80***	70.02±10.00***
	/	Farrerol (0.1 μM, 16 h)	6	148	85.83±6.29***	74.50±7.57***	53.86±7.78***
	/	Farrerol (0.2 μM, 16 h)	3	58	41.55±2.62***	39.79±3.33	32.92±3.39
	/	RS-1 (5 μM, 16 h)	2	57	72.95±5.31	52.56±0.38	33.63±7.54
	/	RS-1 (10 μM, 16 h)	5	117	80.53±10.37*	67.61±16.87*	46.67±12.78
	/	RS-1 (10 μM, 22 h)	2	44	91.37±3.37**	77.47±1.47**	63.58±0.42***
	/	RS-1 (15 μM, 16 h)	2	53	63.65±5.32	41.88±3.95	29.38±8.55
IVF	si/sgControl	Control-DMSO (1000×, 16 h)	2	60	100±0.00	100±0.00	98.28±1.72
	si*Uchl3* (20 μM)+sg*Uchl3* (50 ng/μL)	DMSO (1000×, 16 h)	2	56	98.21±1.79	96.43±0.00	91.07±1.79
SCNT	si/sgControl	Control-DMSO (1000×, 16 h)	3	59	69.59±2.98	50.79±6.54	42.36±0.35
	si*Uchl3* (20 μM)+sg*Uchl3* (50 ng/μL)	DMSO (1000×, 16 h)	3	58	65.33±6.23	41.92±8.43	40.69±7.67
	si/sgControl	Farrerol (0.05 μM, 16 h)	2	39	90.08±4.37*	80.16±8.73*	69.44±2.78***
	si*Uchl3* (20 μM)+sg*Uchl3* (50 ng/μL)	Farrerol (0.05 μM, 16 h)	4	89	70.00±5.11	63.34±5.37	52.78±2.78**

IVF, *in vitro* fertilization; SCNT, somatic cell nuclear transfer

Statistical significance was calculated with a two-tailed unpaired t-test. *P<0.05; **P<0.01; ***P<0.001 as compared with the corresponding control group.

Supplementary Table 2 H3K27me3-positive-ICM blastocysts derived from IVF and SCNT embryos

Embryo type	mRNA injected	Treatment	No. of blastocysts examined	No. of H3K27me3-positive-ICM blastocysts	Ratio
NF blastocyst (Zhang et al.) [69]	N/A	N/A	34	34	100%
IVF blastocyst	/	DMSO (1000×)	15	15	100%
IVF blastocyst	/	Farrerol (0.05 μM)	17	17	100%
SCNT blastocyst (Zhang et al.) [69]	N/A	N/A	42	1	2.38%
SCNT blastocyst	/	DMSO (1000×)	14	1	7.14%
SCNT blastocyst	/	Farrerol (0.05 μM)	24	12	50%
SCNT blastocyst	/	RS-1 (10 μM)	13	2	15.38%
SCNT blastocyst	si/sgControl	Farrerol (0.05 μM)	16	7	43.75%
SCNT blastocyst	si*Uchl3* (20 μM)+sg*Uchl3* (50 ng/μL)	Farrerol (0.05 μM)	20	4	20%

IVF, *in vitro* fertilization; SCNT, somatic cell nuclear transfer; N/A, not applicable

69. Zhang M, Wang F, Kou Z, Zhang Y, Gao S. Defective chromatin structure in somatic cell cloned mouse embryos. *The Journal of biological chemistry* **284**, 24981-24987 (2009).

Supplementary Table 3 In vivo development of SCNT embryos

mRNA injected	Treatment	No. of replicates	No. of 2-cell or 4-cell embryos transferred	No. of pups (% per ET)
/	Control-DMSO (1000×)	8	998	0.2±0.3
/	Farrerol (0.05 μM)	5	372	2.0±0.8***
/	Farrerol (0.1 μM)	4	152	4.0±2.5**
si/sg*Uchl3*	Farrerol (0.05 μM)	3	173	0***

ET, embryo transfer.

Statistical significance was calculated with a two-tailed unpaired t-test. **P<0.01; ***P<0.001 as compared with the control group.
