# Supplementary material for: Farrerol directly activates the deubiqutinase UCHL3 to promote DNA repair and reprogramming when mediated by somatic cell nuclear transfer
Source: Nat Commun. 2023 Apr 3;14:1838. doi: 10.1038/s41467-023-37576-9 (PMC10070447; doi:10.1038/s41467-023-37576-9)
Supplement: Supplementary file 3 — Description of Additional Supplementary Files [file 41467_2023_37576_MOESM3_ESM.pdf]

Description of Additional Supplementary Files

File Name: Supplementary Data 1
Description: Summary of potential farrerol binding peptides and retrieved proteins discovered by LiP-SMap.
