# Supplementary material for: Farrerol directly activates the deubiqutinase UCHL3 to promote DNA repair and reprogramming when mediated by somatic cell nuclear transfer
Source: Nat Commun. 2023 Apr 3;14:1838. doi: 10.1038/s41467-023-37576-9 (PMC10070447; doi:10.1038/s41467-023-37576-9)
Supplement: Supplementary file 5 — Reporting Summary [file 41467_2023_37576_MOESM5_ESM.pdf]

nature portfolio

Corresponding author(s): Ying Jiang

Last updated by author(s): Mar 8, 2023

Reporting Summary

Nature Portfolio wishes to improve the reproducibility of the work that we publish. This form provides structure for consistency and transparency in reporting. For further information on Nature Portfolio policies, see our Editorial Policies and the Editorial Policy Checklist.

Statistics

For all statistical analyses, confirm that the following items are present in the figure legend, table legend, main text, or Methods section.

n/a	Confirmed	
☐	☒	The exact sample size (*n*) for each experimental group/condition, given as a discrete number and unit of measurement
☐	☒	A statement on whether measurements were taken from distinct samples or whether the same sample was measured repeatedly
☐	☒	The statistical test(s) used AND whether they are one- or two-sided<br>*Only common tests should be described solely by name; describe more complex techniques in the Methods section.*
☒	☐	A description of all covariates tested
☐	☒	A description of any assumptions or corrections, such as tests of normality and adjustment for multiple comparisons
☐	☒	A full description of the statistical parameters including central tendency (e.g. means) or other basic estimates (e.g. regression coefficient) AND variation (e.g. standard deviation) or associated estimates of uncertainty (e.g. confidence intervals)
☐	☒	For null hypothesis testing, the test statistic (e.g. *F*, *t*, *r*) with confidence intervals, effect sizes, degrees of freedom and *P* value noted<br>*Give P values as exact values whenever suitable.*
☒	☐	For Bayesian analysis, information on the choice of priors and Markov chain Monte Carlo settings
☒	☐	For hierarchical and complex designs, identification of the appropriate level for tests and full reporting of outcomes
☒	☐	Estimates of effect sizes (e.g. Cohen's *d*, Pearson's *r*), indicating how they were calculated

Our web collection on statistics for biologists contains articles on many of the points above.

Software and code

Policy information about availability of computer code

Data collection	Phenix software (v8.3) , BD FACSVerse (BD Biosciences, San Jose, CA), ABI7500 Real-Time PCR System (Applied Bio Systems), Western images by chemiluminescence using Tanon 4600, SpectraMax iD3, Leica TCS SP8 confocal laser microscopy system (Leica Microsystems, Buffalo Grove, IL), XtaLAB Synergy Custom FRX (Japan).
Data analysis	FlowJo7.6 software (Ashland, OR), Graph Pad Prism 9.0 software, Cometscore software (casplab_1.2.3b2), Biacore T200 Evaluation Software 3.0 (GE Healthcare), RNA-Seq: Sequencing reads were first trimmed to remove adapters and then mapped to the annotated mouse reference genome (UCSC mm10) using HISAT2 (v2.2.1) and sorted by SAMtools (v1.12). The reads were counted by TEcount (v2.2.1). The counts were normalized using variance stabilizing transformations methods by DESeq2 (v1.24.0) and subsequently used for detecting differentially expressed genes (DEGs). The gene expression level was quantified as fragments per kilobase per million mapped reads (FPKM) using StringTie (v2.1.7) and the R package Ballgown (v2.16.0).

For manuscripts utilizing custom algorithms or software that are central to the research but not yet described in published literature, software must be made available to editors and reviewers. We strongly encourage code deposition in a community repository (e.g. GitHub). See the Nature Portfolio guidelines for submitting code & software for further information.

Data

Policy information about availability of data

All manuscripts must include a data availability statement. This statement should provide the following information, where applicable:

- Accession codes, unique identifiers, or web links for publicly available datasets
- A description of any restrictions on data availability
- For clinical datasets or third party data, please ensure that the statement adheres to our policy

The RNA-seq data generated in this study have been deposited in the Genome Sequence Archive in National Genomics Data Center, China National Center for Bioinformation/Beijing Institute of Genomics, Chinese Academy of Sciences, under accession code CRA007477 [https://bigd.big.ac.cn/gsa/browse/CRA007477]. The target gene list of CDX2 and NANOG is from CHEA Transcription Factor Targets, which can be accessed through Harmonizome [https://maayanlab.cloud/Harmonizome/]. Sequencing reads were first trimmed to remove adapters and then mapped to the annotated mouse reference genome (UCSC mm10, https://genome.ucsc.edu/) using HISAT2 (v2.2.1) and sorted by SAMtools (v1.12). The mass spectrometry proteomics data (including raw MS data) in this study have been deposited to the ProteomeXchange Consortium via the PRIDE partner repository with the dataset identifier PXD036308. The UCHL3-farrerol complex structure used herein is available in the PDB database under accession code 7YV4 [DOI: https://doi.org/10.2210/pdb7YV4/pdb]. Source data are provided with this paper.

Human research participants

Policy information about studies involving human research participants and Sex and Gender in Research.

Reporting on sex and gender	This study did not involve any human participants.
Population characteristics	This study did not involve any human participants.
Recruitment	This study did not involve any human participants.
Ethics oversight	This study did not involve any human participants.

Note that full information on the approval of the study protocol must also be provided in the manuscript.

Field-specific reporting

Please select the one below that is the best fit for your research. If you are not sure, read the appropriate sections before making your selection.

☒ Life sciences ☐ Behavioural & social sciences ☐ Ecological, evolutionary & environmental sciences

For a reference copy of the document with all sections, see nature.com/documents/nr-reporting-summary-flat.pdf

Life sciences study design

All studies must disclose on these points even when the disclosure is negative.

Sample size	Sample sizes were chosen based on our previous experience to obtain statistical significance and reproducibility. Exact sample size are included in the paper.
Data exclusions	No exclusion of data was made.
Replication	All experimental data was reliably reproduced in multiple independent experiments as indicated in the figure legends.
Randomization	Randomization was not part of the study design. In vitro assay samples were assigned to experimental in a non-biased way.
Blinding	The investigators were blinded during data collection and analysis where possible. This included immunofluorescence assay data collection for the quantification of RAD51 foci and gammaH2AX positive cells in different types of cells, as well as immunofluorescence assay showing H3K27Me3 intensity. Blinding was not practically feasible to carry out during the other experiments in this study, and it would not be expected to impact the results.

Reporting for specific materials, systems and methods

We require information from authors about some types of materials, experimental systems and methods used in many studies. Here, indicate whether each material, system or method listed is relevant to your study. If you are not sure if a list item applies to your research, read the appropriate section before selecting a response.

Materials & experimental systems

n/a	Involved in the study
☐	☒ Antibodies
☐	☒ Eukaryotic cell lines
☒	☐ Palaeontology and archaeology
☐	☒ Animals and other organisms
☒	☐ Clinical data
☒	☐ Dual use research of concern

Methods

n/a	Involved in the study
☒	☐ ChIP-seq
☐	☒ Flow cytometry
☒	☐ MRI-based neuroimaging

Antibodies

Antibodies used

Primary antibodies used in this study were as follows: β-TUBULIN (Bioworld, Cat. # AP0064, WB: 1:2000). UCHL3 (Proteintech, Cat. # 12384-1-AP, WB: 1000), HA (Cell Signaling Technology, Cat. # 2367, WB: 1:1000), HA (Cell Signaling Technology, Cat. # C29F4, IF: 1:800), Flag (Abclonal, Cat. # AE005, WB: 1:5000), RAD51 (Abcam, Cat. # ab176458, WB: 1:10000, IF: 1:1000 for cells, IF: 1:200 for embryos), GFP (Abclonal, Cat. # AE012, WB: 1:5000), γH2AX (Cell Signaling Technology, Cat. # 9718S, IF: 1:500), CDX2 (BioGenex, Cat. # MU392A-5UC, IF: 1:200), NANOG (Abcam, Cat. # ab80892, IF: 1:200), H3K27me3 (Diagenode, Cat. # C15410195, IF: 1:200), GATA6 (R&D Systems, Cat. # AF1700-SP, IF: 1:200), 5-Methylcytosine (5-mC) (Active Motif, Cat. # 61255, IF: 1:200). Molecular Probes DAPI (4',6 Diamidino 2 Phenylindole) (Thermo Scientific, Cat. # D3571, IF: 1:1000).
Secondary antibodies were used: donkey anti-Rabbit 488 (Invitrogen, Cat. # A21206, IF: 2 μg/mL) and donkey anti-mouse 594 (Fisher Scientific, Cat. # A21203, IF: 4 μg/mL).

Validation

β-TUBULIN antibody (Bioworld, Cat. # AP0064) was validated by WB analysis of A549 whole cell lysate (20 μg), PC12 whole cell lysate(20 μg), BV2 whole cell lysate (20 μg), the Kidney tissue lysate of Mouse (20 μg) and the Prostate tissue lysate of Rat (20 μg) (validation information on the manufacturer's website)
UCHL3 antibody (Proteintech, Cat. # 12384-1-AP) was vailidated by WB of cell extracts in HEK293 cells (in this paper), BxPC-3 cells(PMID: 31598398), U2OS cells (PMID: 27941124, 30559450), and in non-small cell lung cancer tissues and cell lines (PMID: 34016790)
HA antibody (Cell Signaling Technology, Cat. # 2367) was validated by WB analysis of extracts from COS cells untransfected, expressing HA-tagged Akt3 or expressing HA-tagged Estrogen Receptor (ER). (validation information on the manufacturer's website)
HA antibody (Cell Signaling Technology, Cat. # 2367) was validated by confocal immunofluorescence analysis of COS cells, transfected with an HA-tagged protein or mock-transfected. (validation information on the manufacturer's website)
Flag antibody (Abclonal, Cat. # AE005) was vailidated by WB of cell extracts of T. gondii RH strain tachyzoites tag endogenous ROP18 at the C-terminus with eGFP-FLAG (PMID: 29104504).
RAD51 antibody (Abcam, Cat. # ab176458) was vailidated by WB of cell extracts in mouse ES cell (PMID: 30024881) and HeLa cell extract (validation information on the manufacturer's website).
RAD51 antibody (Abcam, Cat. # ab176458) was validated by immunofluorescence detection of Rad51 foci formation after X-ray irradiation in GM0637 cells (validation information on the manufacturer's website).
GFP antibody (Abclonal, Cat. # AE012) was vailidated by WB of cell extracts in HEK293(PMID: 33473130).
γH2AX antibody (Cell Signaling Technology, Cat. # 9718S) was validated by immunofluorescence detection of xenograft tumors irradiated with 7 Gy γ-irradiation (PMID: 29859297), and AGS or MKN28 cells (PMID: 30082912)
CDX2 antibody (BioGenex, Cat. # MU392A-5UC) was validated by immunofluorescence detection of WT/WT and L80A/L80A Oct4 ESCs expressing Cdx2-ERT2-RFP cultured under the TSC culture condition without or with tamoxifen(PMID: 35171666).
NANOG antibody (Abcam, Cat. # ab80892) was validated by immunofluorescence detection of ESCs cultured in 2i (PMID: 26871632) and Equine MAB- or MSC-iPSCs (PMID: 26771353).
H3K27me3 antibody (Diagenode, Cat. # C15410195) was validated by immunofluorescence detection of betaEedKO or control islets (PMID: 29754954) and NIH3T3 cells (validation information on the manufacturer's website).
GATA6 antibody (R&D Systems, Cat. # AF1700-SP) was validated by immunofluorescence detection of single, half or double embryos after 48 h in culture (PMID: 27857135), and Nanog-rescue cells cultured in LIF + FCS (PMID: 31204172)
5-Methylcytosine (5-mC) antibody (Active Motif, Cat. # 61255) was validated by dot blot (validation information on the manufacturer's website) and IHC in rat or human mesotheliomas (PMID: 27129173).

Eukaryotic cell lines

Policy information about cell lines and Sex and Gender in Research

Cell line source(s)

Human embryonic kidney epithelial cells (HEK293, ATCC, CRL-1573), HCA2-hTERT cells (an immortalized foreskin fibroblast cell line) and HCA2-hTERT-H15c cells were originally from Vera Gorbunova's lab (University of Rochester, Rochester, NY).

Authentication

HEK293 cell line authentication was performed by ATCC; HCA2-hTERT cells and HCA2-hTERT-H15c cells were authenticated as described in Mao et al, DNA repair, 2008 (DOI:10.1016/j.dnarep.2008.06.018).

Mycoplasma contamination

All cells were mycoplasma-free with regular checks performed by PCR.

Commonly misidentified lines
(See ICLAC register)

No commonly misidentified cell lines were used in this study.

Animals and other research organisms

Policy information about studies involving animals; ARRIVE guidelines recommended for reporting animal research, and Sex and Gender in Research

Laboratory animals	Species: Mus musculus domesticus<br>Strain: MII oocytes were obtained from eight- to 10-week-old BDF1 (C57BL/6n×DBA2) mice; sperm were collected from the cauda epididymis of adult PWK/PhJ male mice or BDF1 male mice; cumulus cells from eight- to 10-week-old BDF1 or BPF1 (C57BL/6n×PWK/PhJ) female mice were used as nuclear donors; 2-cell embryos were transferred into the oviducts of embryonic day 0.5 (E0.5) eight- to 12-week-old pseudopregnant female ICR mice.
Wild animals	No wild animals were used in this study.
Reporting on sex	MII oocytes were obtained from female BDF1 (C57BL/6n×DBA2) mice; sperm were collected from the cauda epididymis of adult PWK/PhJ male mice or BDF1 male mice; cumulus cells from BDF1 or BPF1 (C57BL/6n×PWK/PhJ) female mice were used as nuclear donors; 2-cell embryos were transferred into the oviducts pseudopregnant female ICR mice.
Field-collected samples	No field-collected samples were used in this study.
Ethics oversight	All mice were provided with free access to food and water. All experiments were performed in accordance with the University of Health Guide for the Care and Use of Laboratory Animals and were approved by the Biological Research Ethics Committee of Tongji University (TJAB04021104).

Note that full information on the approval of the study protocol must also be provided in the manuscript.

Flow Cytometry

Plots

Confirm that:

☒ The axis labels state the marker and fluorochrome used (e.g. CD4-FITC).

☒ The axis scales are clearly visible. Include numbers along axes only for bottom left plot of group (a 'group' is an analysis of identical markers).

☒ All plots are contour plots with outliers or pseudocolor plots.

☒ A numerical value for number of cells or percentage (with statistics) is provided.

Methodology

Sample preparation	HCA-hTERT-H15c and mES cells were trypsinized and resuspended in PBS solution.
Instrument	BD FACSVerse (BD Biosciences, San Jose, CA).
Software	FlowJo7.6 software (Ashland, OR).
Cell population abundance	10 million per mL.
Gating strategy	510-40 nm for GFP positive cells/610-20 nm for DsRed positive cells.

☒ Tick this box to confirm that a figure exemplifying the gating strategy is provided in the Supplementary Information.
